# Impaired functional connectivity of the hippocampus in translational murine models of NMDA-receptor antibody associated neuropsychiatric pathology

**DOI:** 10.1038/s41380-023-02303-9

**Published:** 2023-10-24

**Authors:** Joseph Kuchling, Betty Jurek, Mariya Kents, Jakob Kreye, Christian Geis, Jonathan Wickel, Susanne Mueller, Stefan Paul Koch, Philipp Boehm-Sturm, Harald Prüss, Carsten Finke

**Affiliations:** 1 Department of Neurology and Experimental Neurology, Charité – Universitätsmedizin Berlin, Berlin, Germany; 2 Neurocure Cluster of Excellence, NeuroCure Clinical Research Center, Charité – Universitätsmedizin Berlin, Berlin, Germany; 3 German Center for Neurodegenerative Diseases (DZNE) Berlin, Berlin, Germany; 4 Berlin Institute of Health at Charité – Universitätsmedizin Berlin, Charitéplatz 1, 10117 Berlin, Germany; 5 Section of Translational Neuroimmunology, Hans Berger Department of Neurology, Jena University Hospital, Jena, Germany; 6 Neurocure Cluster of Excellence, Core Facility 7 T Experimental MRIs, Charité – Universitätsmedizin Berlin, Berlin, Germany; 7 Berlin Center for Stroke Research, Charité – Universitätsmedizin Berlin, Berlin, Germany; 8 Humboldt-Universität zu Berlin, Berlin School of Mind and Brain, Berlin, Germany

**Keywords:** Neuroscience, Diagnostic markers

## Abstract

Decreased hippocampal connectivity and disruption of functional networks are established resting-state functional MRI (rs-fMRI) features that are associated with neuropsychiatric symptom severity in human anti-N-methyl-D-aspartate receptor (NMDAR) encephalitis. However, the underlying pathophysiology of NMDAR encephalitis remains poorly understood. Application of patient-derived monoclonal antibodies against the NR1 (GluN1) subunit of the NMDAR now allows for the translational investigation of functional connectivity in experimental murine NMDAR antibody disease models with neurodevelopmental disorders. Using rs-fMRI, we studied functional connectivity alterations in (1) adult C57BL/6 J mice that were intrathecally injected with a recombinant human NR1 antibody over 14 days (*n* = 10) and in (2) a newly established mouse model with *in utero* exposure to a human recombinant NR1 antibody (NR1-offspring) at the age of (2a) 8 weeks (*n* = 15) and (2b) 10 months (*n* = 14). Adult NR1-antibody injected mice showed impaired functional connectivity within the left hippocampus compared to controls, resembling impaired connectivity patterns observed in human NMDAR encephalitis patients. Similarly, NR1-offspring showed significantly reduced functional connectivity in the hippocampus after 8 weeks, and impaired connectivity in the hippocampus was likewise observed in NR1-offspring at the age of 10 months. We successfully reproduced functional connectivity changes within the hippocampus in different experimental murine systems that were previously observed in human NMDAR encephalitis patients. Translational application of this method within a combined imaging and histopathological framework will allow future experimental studies to identify the underlying biological mechanisms and may eventually facilitate non-invasive monitoring of disease activity and treatment responses in autoimmune encephalitis.

## Introduction

Anti-N-methyl-D-aspartate receptor (NMDAR) encephalitis is an autoimmune disorder caused by autoantibodies targeting the NR1 (GluN1) subunit of the NMDAR. Patients present with neuropsychiatric symptoms including decreased levels of consciousness, seizures, behavioral changes, psychosis, catatonia and cognitive deficits [1]. Some patients initially have isolated psychiatric symptoms which can cause misdiagnosis as primary psychiatric disorder [2, 3]. Despite the often severe clinical course, routine brain MRI yields no abnormalities in most patients and therefore provides only limited diagnostic value [4, 5]. By contrast, resting-state functional MRI (rs-fMRI) studies revealed disruption of functional connectivity of the hippocampus with the anterior default mode network (DMN) that correlated with memory performance [6]. Recent large-scale network analyses confirmed these findings and showed decoupling of the medial temporal lobe network from the DMN. Notably, functional decoupling correlated with the severity of memory deficits and disruption of fronto-parietal networks related to psychiatric schizophrenia-like symptoms encompassing catatonia, hallucinations, delusions, and thought disorders [7]. In contrast, another recent rs-fMRI based analysis in seventeen patients with NMDAR encephalitis also reported increased functional connectivity of the posterior cingulate cortex with several brain regions including lingual gyrus, fusiform gyrus, calcarine and cuneus [8]. Together, these observations suggest that NMDAR antibody-associated psychiatric and neurological symptoms are linked to disturbance of functional brain networks [6, 7]. Predominant antibody-mediated dysfunction of the hippocampus is further substantiated by localized atrophy of hippocampal subfields [9]. These previous findings are in line with the fact that the hippocampal formation contains the highest density of NMDARs in the brain [10]. Indeed, both the exact pattern of NMDAR antibody exposure and impaired functional connectivity remain elusive and can only partially be addressed by studies in humans.

Recently, several mouse models of NMDAR encephalitis have been developed [11–15]. We have shown that monoclonal human cerebrospinal fluid-derived antibodies against the NR1 subunit of the NMDAR derived from NMDAR encephalitis patients have direct pathogenicity. Monoclonal antibodies against NR1 caused neuronal surface receptor downregulation and subsequent disruption of synaptic NMDAR currents [16, 17]. Furthermore, injection of monoclonal NR1 antibodies in pregnant maternal mice with subsequent fetal *in utero* exposure resulted in a neurodevelopmental disorder in the offspring [18]. This neurodevelopmental disease model is characterized by long-lasting neuropathological effects reflected by increased postnatal mortality, hyperactivity, lower anxiety and impaired sensorimotor gating. These behavioral abnormalities are complemented by abnormalities in structural MRI with reduced volumes of cerebellum, midbrain and brainstem. Observations in this disease model indicate diaplacentally transferred NR1 antibodies to act as potential contributors transferred via the not fully developed blood-brain-barrier, leading to interference of antibodies with fetal development. These findings were substantiated by the simultaneously reported retrospective substudy on IgG autoreactivity in 120 human mothers of children with psychiatric disorders [18]. NR1 antibody measurements in mothers of children with psychiatric disorders ranging from autism spectrum disorders and bipolar disorders to schizophrenia yielded higher antibody levels when compared with mothers from healthy children. Materno-fetal transfer of neuropathological antibodies that are present during pregnancy, i.e. materno-fetal autoantibodies, may therefore eventually lead to a broader spectrum of behavioral abnormalities found in common psychiatric diseases. In a related study that investigated murine fetal *in utero* exposure to patient NMDAR IgG, brain changes such as thinning of cortical layers alongside depressive-like behavior, poor motor coordination, and impaired social-spatial memory were likewise evident. Symptoms occurred directly after birth during the first postnatal month. However, symptoms and developmental alterations in offspring mice were only present during the early stages after birth, and were completely reversible until adulthood [19]. Collectively, both disease models with in utero exposure to either monoclonal NR1 antibodies or patient NMDAR IgGs show pathological effects to occur during early neuronal and behavioral development in offspring mice. Yet, it remains unclear to which extent in utero exposure to NMDAR antibodies may lead to long-term neurodevelopmental or even neurodegenerative changes.

Recent advances in the acquisition and analysis of functional MRI data in mice now allow for robust and reproducible analysis of functional connectivity alterations and large-scale network dysfunction in mouse models of neurological and psychiatric disorders [20–22]. This provides the unique opportunity to not only study the effect of NMDAR antibody exposure on the functional connectivity, but also to compare the connectivity changes from these animal models with the functional network alterations observed in human patients. Given that potential novel interventional strategies are preclinically first evaluated in mice before being tested in humans, studying the correlation between NMDAR antibody-mediated clinical and imaging features in mice may help to elucidate disease-specific markers that are related to the neuronal dysfunction. Ultimately, this may enable translation of findings in mouse models to human research at the interface of pre-clinical and clinical research prior to phase I and phase II clinical trials.

Here, we performed 7 Tesla (T) rs-fMRI investigations in mice that were injected with human recombinant NR1 antibodies and in the offspring of a recently established murine model with *in utero* exposure to NR1 antibodies characterized by a neurobehavioural disorder. We hypothesized that (i) functional connectivity changes of the hippocampus known from human NMDAR encephalitis are similarly observable in NR1 antibody mouse models and that (ii) functional connectivity alterations are present in antibody-mediated neurodevelopmental brain disorders.

## Materials and methods

### Animal experiments

Animal experiments were carried out in accordance with the Animal Research: Reporting of In Vivo Experiments (ARRIVE) guidelines [23], the EU Directive (2010/63/EU) for animal experiments, and were approved by the local ethics committee for Animal Welfare (Landesamt für Gesundheit und Soziales [LaGeSO], Berlin, G0175/15; Thuringian state authorities, UKJ-17-053). No randomization was used for the assignment of mice to their specific cohort.

**Fig. 1:**
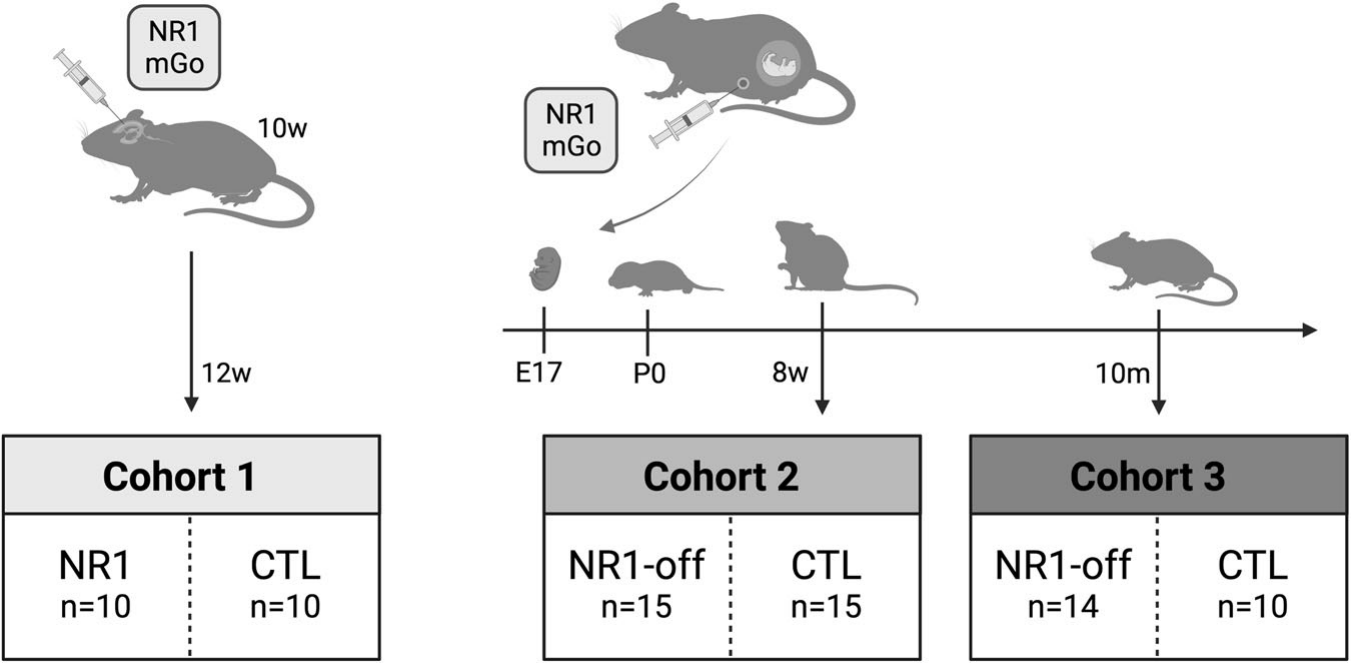
Flowchart of experimental design and cohort selection. Cohort 1: Ten mice were directly exposed to NR1 antibodies at the age of 10 weeks with bilateral infusion of human monoclonal IgG1 antibody into the lateral ventricles using implanted catheters connected to osmotics pumps. Ten mice received human monoclonal IgG1 antibody that is non-reactive to brain tissue (mGo) into the lateral ventricles and served as controls. MR investigation of cohort 1 was performed at the age of 12 weeks. Cohort 2 and 3: Pregnant 8- to 10-week-old mouse dams were injected intraperitoneally with either human monoclonal NR1 antibody or antibody that is non-reactive to brain tissue (mGo), at gestational days E13 and E17. Twenty-nine mice with prior *in utero* exposure to human NR1 antibody were raised according to a previously published protocol (NR1-offspring) and twenty-five offspring mice from maternal animals exposed to intraperitoneally injected human mGo antibody served as controls. Offspring mice were investigated either at the age of 8 weeks (cohort 2; NR1-offspring: *n* = 15; controls: *n* = 15) or at the age of 10 months (cohort 3; NR1-offspring: *n* = 14; controls: *n* = 10). Offspring mice from cohort 2 and cohort 3 were identical with a subproportion of the offspring mice that were investigated elsewhere [18]. NR1 = NR1-reactive human monoclonal IgG1 antibody; mGo = monoclonal IgG1 antibody that is non-reactive to brain tissue (control clone: #mGO53); NR1-off = NR1-offspring cohorts; CTL = controls.

Ten mice were directly exposed to NR1 antibodies during adulthood (NR1; cohort 1; Fig. 1). These C57BL/6 J mice (age of 10 weeks) received bilateral infusion of 200 µg human monoclonal IgG1 antibody (high-affinity NR1-reactive [amino-terminal domain] IgG1 clone: #003-102 [*n* = 10, “NR1”]; NR1 protein binding constant half-maximal concentration c_50_: 1.16) [16, 24] in PBS for 14 days into the lateral ventricles using implanted catheters connected to osmotics pumps following established protocols [25–27]. The association constant for our NR1-reactive IgG1 clone used herein is not available as surface plasmon resonance testing has not been performed using conformational NR1 protein together with our monoclonal NR1 antibodies. However, we previously performed measurement of binding strength to characterize the NR1 antibody (see information on binding constant above) [24]. Ten mice received human monoclonal IgG1 antibody that is non-reactive to brain tissue (control clone: #mGO53) [16, 28] into the lateral ventricles via implanted catheters and served as NR1-controls. MR investigation of NR1 mice and their respective controls was performed at the age of 12 weeks (see Fig. 1).

Additionally, 29 mice (female *n* = 12; male *n* = 17) with prior *in utero* exposure to human recombinant NR1 antibody [16, 24] were raised according to a previously published protocol (NR1-offspring; cohorts 2 and 3; Fig. 1) [18]. In short, 8- to 10-week-old pregnant C57BL/6 J mice were injected intraperitoneally, at gestational days E13 and E17, at each timepoint with 240 μg of human monoclonal IgG1 antibody #003-102. Twenty-five offspring mice (female *n* = 14; male *n* = 11) from maternal animals exposed to intraperitoneally injected human monoclonal IgG1 antibody that is non-reactive to brain tissue (control clone: #mGO53) [16, 28] (Fig. 1) served as controls for cohorts 2 and 3. All offspring mice were housed in treatment-mixed groups of 2-5 animals of both sexes and were investigated either at the age of 8 weeks (cohort 2; NR1-offspring: *n* = 15; controls: *n* = 15) or at the age of 10 months (cohort 3; NR1-offspring: *n* = 14; controls: *n* = 10) (see Fig. 1). Offspring mice from cohort 2 and cohort 3 were identical with a subproportion of equivalent sample size of the offspring mice that were investigated and described in detail with regards to antibody distribution, behavioral abnormalities and regional MRI brain volume changes in a previous report [18].

### MRI data acquisition and quality control

Anesthesia was achieved using 1.5–2% isoflurane in a 70:30 nitrous oxide:oxygen mixture. Before start of the resting-state functional MRI (rs-fMRI) scan, isoflurane levels were reduced to 1.2 ( + /- 0.3) % until animals breathed regularly at higher rate of 160 ( + /-25)/min. This process took 5–7 min and body temperature and respiration rate were monitored with MRI compatible equipment (Small Animal Instruments Inc., Stony Brook, NY). 2D echo-planar imaging (EPI) rs-fMRI images were acquired (repetition time (TR) = 1000 ms; echo time (TE) = 13 ms; flip angle (FA) = 50°; 300 repetitions; 16 axial slices with slice thickness = 0.75 mm; field of view (FOV) = 19.2 ×12.0 mm²; image matrix = 128 ×80; number of averages = 1) on a 7 Tesla (T) MR scanner (Bruker Biospec, Ettlingen, Germany) and a transmit/receive mouse cryoprobe (Bruker). Experimenters were blinded to the condition of the animals.

### rs-fMRI data processing and denoising

Processing of rs-fMRI data was modified according to an established rs-fMRI analysis protocol [29]. Rs-fMRI datasets were skull-stripped using linear affine registration of an in-house mouse atlas template based on C57BL/6 J control mice previously studied at the same scanner to individual mouse rs-fMRI images with Functional Magnetic Resonance Imaging of the Brain (FMRIB) Software Library (FSL) Linear Image Registration Tool (FLIRT) [30]. Each skull-stripped 4D dataset was then fed into FMRIB’s Multivariate Exploratory Linear Optimized Decomposition of Independent Components (MELODIC) [31] to perform within-subject spatial independent component analysis (ICA) with automatic dimensionality estimation, using a skull-stripped EPI image of a deliberately chosen subject as anatomical template. Within-subject ICA was performed after removal of the first 10 out of 300 time points of every subject and included in-plane smoothing with a 1.0 ×1.0 mm kernel. Subsequent denoising of single-subject fMRI independent components was performed in accordance with previously described protocols [29, 32] by manual identification of signal and noise based on the threshold spatial maps, the temporal power spectrum, and the time course by one rater (JKu) who was blinded to the clinical phenotype of mice.

### Group ICA and dual regression

Resting state networks common to all mice were identified for each cohort, separately, by use of temporal-concatenation ICA with automatic component estimation as implemented in FSL MELODIC [31, 33]. To facilitate classification, resulting components were displayed as spatial color-coded z-maps onto the Allen Mouse Brain atlas (AMBA; mouse.brain-map.org/static/atlas) after co-registeration with FSL FLIRT. Group comparisons were carried out using dual regression and nonparametric permutation testing (1,000 permutations) with threshold-free cluster enhancement (TFCE) as implemented in FSL *randomise* (p < 0.05, familywise error [FWE]-corrected) [34]. Resultant maps from group analyses were co-registered to AMBA for visualization.

## Results

### Resting State Component Identification

**Fig. 2:**
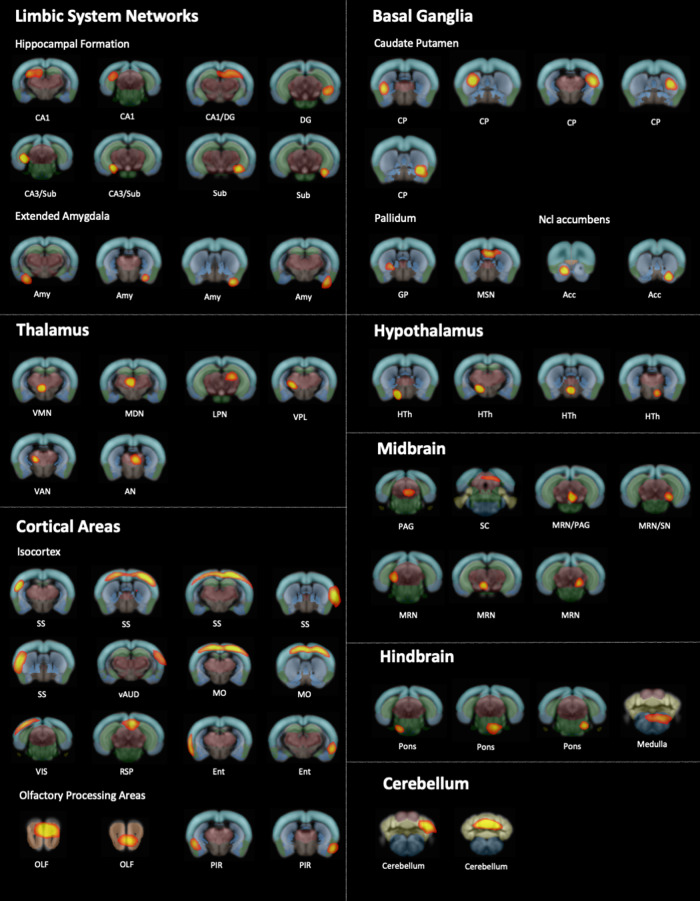
Brain components generated by group independent component analysis (ICA) applied to cohort 2: NR1 offspring at the age of 8 weeks together with 8w-controls. Group independent component analysis (ICA) was applied to all mice from cohort 2, consisting of 15 NR1 offspring at the age of 8 weeks and and 15 controls at the age of 8 weeks. Resting state networks common to all subjects from cohort 2 (*n* = 30) were identified using temporal-concatenation ICA as implemented in Functional Magnetic Resonance Imaging of the Brain Software Library Multivariate Exploratory Linear Optimized Decomposition of Independent Components (FSL MELODIC) [31, 33]. Anatomically plausible components that were generated by group ICA are displayed as spatial color-coded z-maps (red-yellow heat maps) onto the Allen Mouse Brain Atlas (AMBA). Components are arranged according to anatomical mouse brain structures after visual correlation with AMBA brain regions modified after previously published component grouping [29, 71]. Components were found in all main anatomical regions and were subsequently used for group comparison of functional connectivity between NR1 offspring and controls (see Figs. 3–5). Anatomical labels: DG = dentate gyrus; Sub = subiculum; Amy = amygdala; aCing = anterior cingulate area; VMN = thalamic ventromedial nucleus; LGN = thalamic lateral geniculate nucleus; MDN = medial dorsal nucleus; LPN = lateral posterior nucleus; VPL = thalamic ventral posterolateral nucleus; VAN = ventral anterior nucleus; AN = anterior nuclear group of the thalamus; SS = primary somatosensory area; vAUD = ventral auditory area; MO = pimary motor area; VIS = primary and anterolateral visual area; RSP = retrosplenial cortex; Ent = entorhinal area; Ent = lateral part of the entorhinal area; OLF = main olfactory bulb; PIR = piriform area; CP = caudate putamen; GP = globus pallidus; MSN = medial septal nucleus area of pallidum; Acc = nucleus accumbens; HTh = Hypothalamus; PAG = periaqueductal gray; SC = superior colliculus; MRN = midbrain.

Group independent component analysis (ICA) identified canonical functional components with similar anatomical localization in cohort 1 (NR1 and controls; 90 components), cohort 2 (8w-offspring and 8w-controls; 90 components), and cohort 3 (10m-offspring and 10m-controls; 95 components). Component patterns consistently covered common neuroanatomical regions defined by co-registration with the AMBA, including brainstem, hypothalamus, thalamic areas with differentiation of thalamic subnuclei, somatosensory cortical areas, hippocampal formation and basal ganglia regions (Fig. 2). Of note, the identification of functional components within the hippocampal formation allowed for the distinction between CA1-3, dentate gyrus and subiculum correlating with corresponding anatomical AMBA regions. See Table 1 for a comparative overview regarding cohort characteristics and functional connectivity results.

**Table 1:** Cohort Characteristics Overview.

	Adult NR1	8w-offspring	10m-offspring
Cohort	Cohort 1	Cohort 2	Cohort 3
**mode of antibody exposure**	bilateral infusion of NR1 antibody into lateral ventricles	materno-fetal exposure to NR1 antibody	materno-fetal exposure to NR1 antibody
**antibody application route**	intrathecal injection	*in utero* exposure via diaplacental transfer	*in utero* exposure via diaplacental transfer
**NR1 IgG clone**	#003-102	#003-102	#003-102
**age of mice at MR investigation**	12 weeks	8 weeks	10 months
**number of mice (n) vs. controls (n)**	10 vs. 10	15 vs. 15	14 vs. 10
**hippocampal functional connectivity change**	functional connectivity impairment in left dentate gyrus	functional connectivity impairment in left dentate gyrus	functional connectivity impairment in left dentate gyrus and CA3
**previously published behavioral abnormalities**	no behavioral abnormalities. Higher epileptogenic predisposition with increased numbers of convulsive seizures, higher total seizure score, and higher number of epileptic ‘spike’ events [14]	abnormal neuropsychiatric phenotype, including hyperactivity, lower anxiety and impaired sensorimotor gating [17]	abnormal neuropsychiatric phenotype, including hyperactivity, lower anxiety and impaired sensorimotor gating [17]

### Cohort 1: NR1 mice

**Fig. 3:**
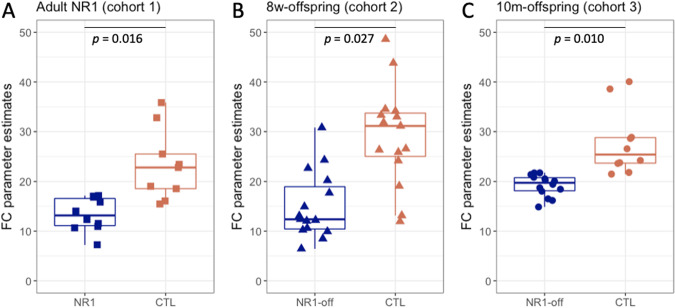
Comparison of hippocampal functional connectivity parameter estimates in NR1 antibody mouse models compared to control mice. Statistical significance was determined using dual regression and nonparametric permutation testing (1,000 permutations) with threshold-free cluster enhancement (TFCE) as implemented in Functional Magnetic Resonance Imaging of the Brain Software Library (FSL *randomise* function (p < 0.05, familywise error [FWE]-corrected) [34] within functionally defined regions of interest. Significantly decreased left hippocampal functional connectivity was observed in **A** adult NR1 mice, **B** NR1-offspring at 8 weeks and **C** NR1-offspring at the age of 10 months compared to their respective control groups.

**Fig. 4:**
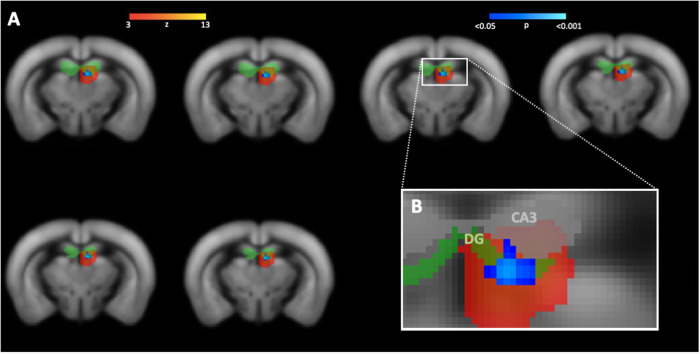
Reduced functional connectivity within hippocampus (dentate gyrus) of NR1 mice compared to controls (cohort 1). **A** Dual regression analysis revealed reduced functional connectivity of adult NR1 mice compared to controls (significantly different area displayed based on p-values in blue-lightblue) in a specific functional component (component area displayed as z-map in red-yellow). **B** Functional component and corresponding area of functional connectivity reduction is affiliated with frontal (but not posterior) parts of the left-hemispheric hippocampus (light-green region of interest) and C shows visual correspondence with the frontal parts of the hippocampal subfield dentate gyrus (green). No significant differences were seen at the middle or posterior portions of the dentate gyrus / hippocampal formation. DG = dentate gyrus.

NR1 mice showed significantly reduced functional connectivity in the left hippocampus in comparison to control mice (Fig. 3A). According to the Allen Mouse Brain atlas (AMBA), the major cluster of reduced functional connectivity (*p* = 0.016) was primarily localized within left dentate gyrus (Fig. 4). No further functional connectivity alterations were observed in NR1 mice compared to controls (see Supplementary Fig. S1).

### Cohort 2: 8w-offspring

**Fig. 5:**
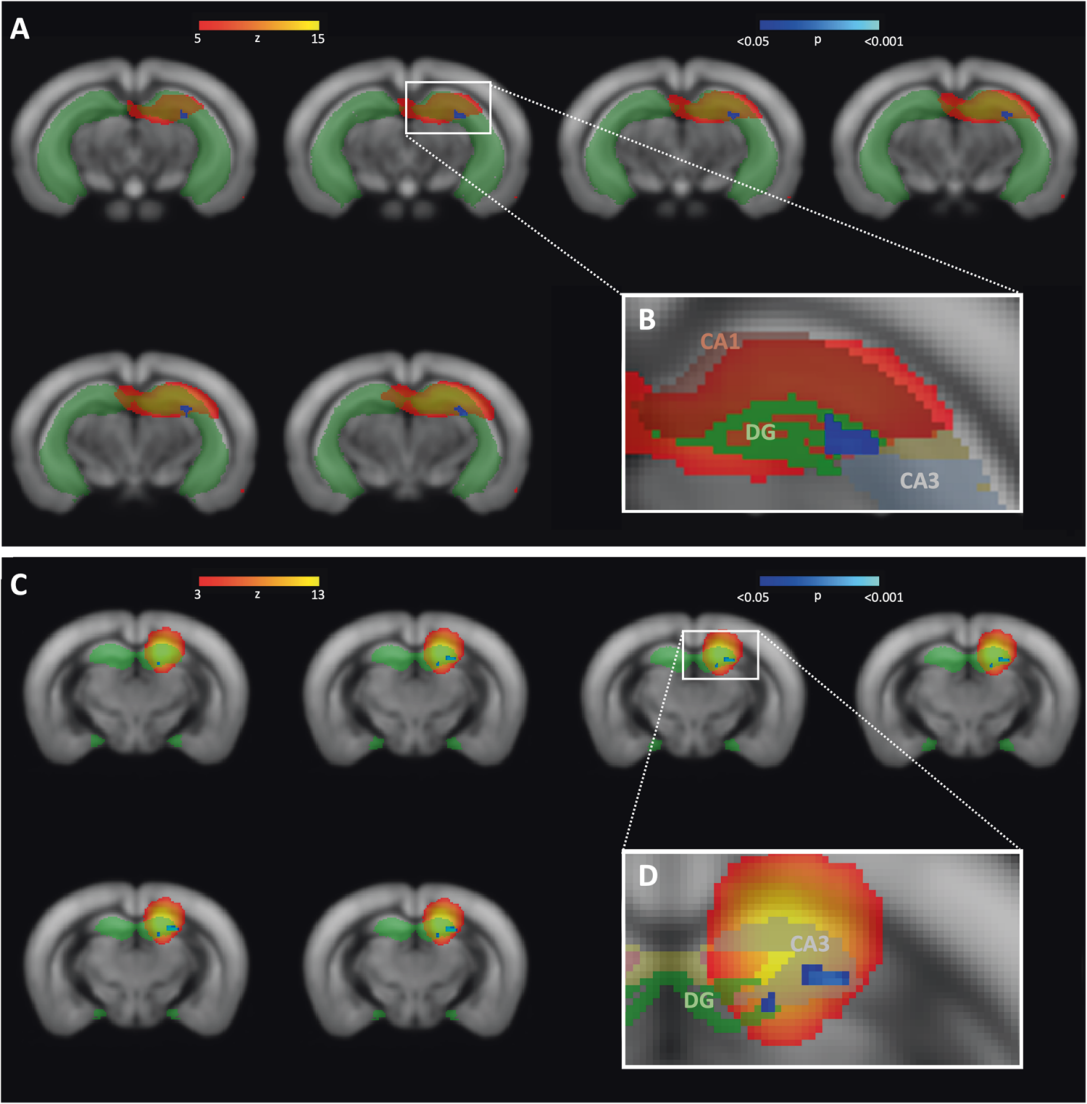
Reduced functional connectivity within hippocampus of NR1 offspring at different stages of age compared to controls (cohort 2 and 3). **A** Dual regression analysis revealed reduced functional connectivity of 8-week-old NR1 offspring with neurodevelopmental disorder compared to controls (significantly different area displayed based on p-values in blue-lightblue) in a specific functional component (component area displayed as z-map in red-yellow) affiliated with left-hemispheric hippocampus (light-green region of interest) and **B** shows visual correspondence with hippocampal subfield with frontal (but not posterior) parts of dentate gyrus (green). **C** Reduced functional connectivity of 10-month-old NR1 offspring is detectable in a functional component corresponding with left-hemispheric hippocampus (light-green region of interest). **D** Visual correspondence of impaired connectivity cluster with hippocampal subfield of dentate gyrus (green) and CA3 (white). DG = dentate gyrus.

Antibody-mediated effects of monoclonal NR1 antibodies in NR1 offspring mouse model investigated in cohorts 2 and 3 on the biomolecular and behavioral level have been previously described in detail at different developmental stages and have recently been published elsewhere [18]. NR1-offspring at the age of 8 weeks exhibited selectively reduced functional connectivity of the left hippocampus (*p* = 0.027) in comparison to control mice (Fig. 3B). Functional connectivity alterations were primarily located in the dentate gyrus according to the AMBA (Fig. 5A and B). No further significant functional connectivity differences between 8w-offspring mice and controls were observed (see Supplementary Fig S2).

### Cohort 3: 10m-offspring

Reduced functional connectivity was observed within the left hippocampus (*p* = 0.010) in NR1-offspring at the age of 10 months compared to control mice (Fig. 3C). Specifically, functional connectivity alterations were located in the dentate gyrus and CA3 (Fig. 5C and D). No further functional connectivity differences between 10m-offspring mice and controls were found (see Supplementary Fig. S3).

### Human NR1 antibodies bound to synaptic structures

**Fig. 6:**
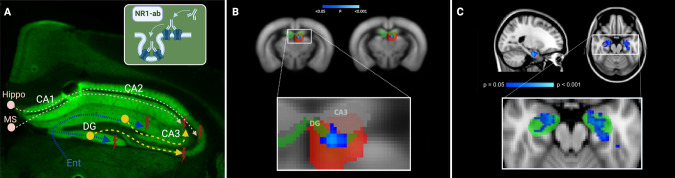
Comparative pathology and MR correlates of impaired hippocampal functional connectivity in human anti-N-methyl-D-aspartate receptor (NMDAR) encephalitis and murine NMDAR antibody disease models. **A** Pathophysiological mechanisms in murine NMDAR encephalitis disease model, drawn in the immunofluorescence staining of a coronal brain section from a mouse with intrathecal NR1 antibody injection of cohort 1. As expected, immunofluorescence on murine brain sections shows characteristic antibody binding of high-affinity NR1-reactive monoclonal IgG1 antibody (clone #003-102) to hippocampal neuropil. This characteristic pattern has been detected in previous works using similar human cerebrospinal fluid NMDAR autoantibodies [16]. Preferential antibody-binding to hippocampal neuropil is explained by high expression of NMDA receptors in this region [10]. Particularly, NR1-subunit of the NMDAR is widely expressed in all parts of the human hippocampal formation, including dentate gyrus, CA1-CA3, subiculum, presubiculum and entorhinal cortex [36] and similar distribution patterns are likewise seen in rodent brains with hippocampal levels of NR1 protein widely exceeding the levels of cortex, olfactory bulb, midbrain and cerebellum [37]. NR1-antibodies lead to degradation of hippocampal NMDAR in dentate gyrus and CA1-CA3. Projection fibers from entorhinal cortex (blue) are entering *stratum moleculare* of dentate gyrus via perforant path. Pyramidal neurons in CA3 receive intrahippocampal projection fibers from dentate gyrus (yellow) and from external fibers from medial septum and contralateral hippocampus (pink). Dysfunction of hippocampal neurons in dentate gyrus and CA3 lead to impairment in the connectivity of hippocampal circuits. **B** Reduced functional connectivity (significantly different area displayed based on p-values in blue-lightblue) within frontal (but not posterior) parts of the hippocampus (hippocampal functional component area displayed as z-map in red-yellow; anatomical area of dentate gyrus displayed in green) of NR1 mice compared to controls (cohort 1). CA3 (gray) is labeled according the Allen Mouse Brain Atlas (AMBA). No significant differences were seen at the middle or posterior portions of the dentate gyrus / hippocampal formation. **C** Reduced functional connectivity (blue) between both the left and the right hippocampus (green area) and the anterior default-mode network in NMDARE patients compared to healthy controls (original picture published in Finke et al. [6]). Ent = entorhinal cortex; NR1-ab = NR1 IgG antibodies; NMDARE = anti-NMDAR encephalitis; MS = medial septum; Hippo = contralateral hippocampus; DG = dentate gyrus; Sub = subiculum.

Hippocampal staining revealed the well-known distribution pattern in pivotal regions of hippocampal connectivity, i.e. dentate gyrus and CA3 region, that receive projections from entorhinal cortex and from medial septum and contralateral hippocampus [35]. Immunofluorescence of mouse brain sections post-MRI in cohort 1 confirmed the strong binding of intrathecally administered human monoclonal NR1 IgG antibodies on hippocampal neuropil on murine brain sections (Fig. 6A).

## Discussion

We observed selectively impaired functional connectivity of the hippocampus in an adult mouse model of anti-N-methyl-D-aspartate receptor (NMDAR) encephalitis. In addition, selective impairment of hippocampal functional connectivity was found in mice with *in utero* exposure to monoclonal NR1 antibodies at two different time points. These findings mirror observations of functional connectivity alterations in human patients with NMDAR encephalitis and are in line with the predominant expression of NMDAR in the hippocampus. As such, our study identifies the first cross-species imaging biomarker in NMDAR encephalitis and provides a promising foundation for translational studies on the pathophysiology and on preclinical treatment evaluation of the disease.

### Comparison of mouse data with human data

In a whole brain functional connectivity analysis in a mouse model of NMDAR encephalitis, we observed a selective disruption of hippocampal connectivity. This pattern is remarkably consistent with findings in human patients: In a sample of 24 NMDAR encephalitis patients, selectively reduced functional connectivity of the hippocampus with the anterior default mode network (DMN) was found. Moreover, the level of this hippocampal connectivity disruption correlated with the severity of memory impairment [6]. Subsequent large-scale network and whole-brain pair-wise connectivity analyses in 43 NMDAR encephalitis patients corroborated these findings. They likewise showed impaired hippocampal connectivity alongside decoupling of medial temporal network and the DMN. In addition, an overall impairment of frontotemporal connections was reported that correlated with memory impairment and schizophrenia-like symptoms including anhedonia, catatonia, hallucinations, delusions, and thought disorders [7]. Our current results of selectively impaired functional connectivity of the hippocampus in mice after passive transfer of NMDAR antibodies indicate (i) the central role of hippocampal dysfunction in the pathophysiology of NMDAR encephalitis, (ii) the validity of the employed NMDAR encephalitis mouse model mirroring previous findings in human patients, (iii) the relevance of materno-fetal NMDAR antibody transmission and (iv) the translational applicability of resting state functional MRI analyses – as will be discussed in the following.

#### Central role of hippocampal dysfunction and NMDAR down-regulation

The predominant vulnerability of the hippocampus in NMDAR encephalitis is in line with the fact that hippocampus contains the highest density of NMDARs in the human brain [10]. Particularly, the NR1 subunit of the NMDAR is widely expressed in all parts of the human hippocampus, including the dentate gyrus, CA1-CA3, subiculum, presubiculum and entorhinal cortex [36]. Similar distribution patterns are seen in rodent brains with hippocampal levels of NR1 protein widely exceeding the levels of cortex, olfactory bulb, midbrain and cerebellum [37]. Similarly, a recent collation of molecular positron emission tomography (PET) data from healthy individuals showed a high expression of NMDAR in the posterior cingulate and the retrosplenial cortex (that also belong to the DMN), but an even higher expression was observed in the hippocampus [[38], *personal communication*]. Recently, a graph theoretical network analysis based on structural connectomes revealed bilateral reductions in hippocampal node strength in NMDAR encephalitis patients [39]. A strong association was found between hippocampal node strength and verbal long-term memory. In line with this, volumetric MRI analyses and structural connectome analyses in human patients with NMDAR encephalitis show localized atrophy of hippocampal subfields and impaired structural hippocampal connectivity that correlated with individual deficits in verbal memory performance [9], lending further support to the concept of predominant hippocampal damage.

Of note, all cohorts investigated herein showed lateralized effects with reduced functional connectivity in the left (but not right) hippocampus. While studies in human patients with NMDAR encephalitis observed bilaterally reduced hippocampal functional connectivity [6, 7], lateralized functional roles of the hippocampus are well established [40]. Indeed, the left-right asymmetry in size, shape, and glutamate receptor expression of murine hippocampal synapses may potentially account for lateralized functional connectivity findings. Synaptic distribution of NMDAR NR2B subunits in the adult mouse hippocampus is asymmetrical between left and right [41, 42] with an absolute NR2B subunit density decrease in the right hippocampus [43]. Accordingly, behavioral investigations in mice showed that selective silencing of the CA3 area of the left hippocampus impaired associative spatial long-term memory, whereas the equivalent manipulation in the right hippocampus did not [44]. Together, these observations might well explain the lateralized effects of impaired hippocampal connectivity in the current study. Future animal studies with larger cohorts are warranted to further assess whether bilateral hippocampal functional connectivity changes are detectable in NR1 antibody mouse models.

However, the clinical syndrome of NMDAR encephalitis clearly extends beyond hippocampal dysfunction and suggests additional cortical and subcortical involvement [45]. This is supported by recent studies showing reduced functional connectivity in distributed large-scale networks, including sensorimotor, frontoparietal, and visual networks [7] and is in line with global metabolic changes observed using PET [46, 47]. A recent study on 17 NMDAR encephalitis patients reported on a pattern with amplitude of low-frequency fluctuation decrease within posterior cingulate gyrus, left precuneus, and bilateral cerebellar regions [8]. In addition, extensive white matter damage assessed by diffusion tensor imaging related to disease severity and cognitive impairment has been previously reported in NMDAR encephalitis patients [6, 48–50]. Moreover, a recent diffusion kurtosis imaging study also reported extensive gray matter microstructural damage in patients with NMDAR encephalitis [51]. Together, these studies indicate brain-wide dysfunction of NMDA receptors, with predominant alteration of hippocampal function. Indeed, NMDAR antibodies were shown to bind throughout the rodent brain - with strong preferential binding in the hippocampus, leading to decreased NMDAR cluster density on neurons [52]. In addition, it was recently found that NMDAR antibodies also alter the function of NMDAR in oligodendrocytes, thus providing a link between antibody-mediated NMDAR dysfunction and widespread white matter damage observed using MRI [53]. In the current study, we did not observe the same widespread network disruption reported in human NMDAR patients but detected selective functional connectivity changes of the hippocampus. Indeed, earlier studies in humans with smaller numbers of patients and similar analysis methods likewise observed functional connectivity alterations restricted to the hippocampus [6]. Recent investigations in larger patient samples [7] and studies that used more elaborate methods, e.g. dynamic functional connectivity analyses [54, 55], identified more widespread functional disruptions. It is therefore conceivable that future studies with larger and further refined animal models, imaging protocols and post-processing methods will be able to detect more widespread functional network changes as predicted by human MRI studies. Furthermore, diffusion tensor imaging studies – ideally in combination with histopathological analyses – are warranted to investigate white matter integrity in NMDAR encephalitis mouse models to advance the understanding of oligodendrocyte pathophysiology in this disease.

Given that binding of human monoclonal NR1 antibodies to NMDARs is sufficient to cause morphological and electrophysiological changes in neurons resulting from NMDAR down-regulation [16], the presence of cerebrospinal fluid (CSF)-derived NR1 antibodies from patients provides a plausible mechanism for the occurrence of neuropsychiatric symptoms observed in NMDAR encephalitis [56]. NMDAR down-regulation is therefore suggested to play the key role in mediating clinical psychiatric symptoms, i.e. psychosis and catatonia, analogous to the established NMDAR hypofunction model in schizophrenia [56]. Persistent blockade of NMDAR in experimental animals recreates the pathologic features of schizophrenia including down regulation of parvalbumin-positive cortical gamma-aminobutyric acid (GABA)ergic neurons, pyramidal neuron dendritic dysgenesis, and reduced spine density [56, 57]. Conditional NMDAR subunit NR1 knockout mice have been used to create animal models similar to those observed in pharmacologically induced animal models of schizophrenia that can be ameliorated by antipsychotic treatment [58]. These experimental findings are complemented by historical observations that NMDAR antagonists mimic the positive and negative symptoms of schizophrenia [56, 59]. Furthermore NMDARs are reduced in unmedicated schizophrenia [60]. In addition, many of the genes associated with schizophrenia are linked to the NMDAR or related synaptic structures [61]. Hence, monoclonal antibodies to the NMDAR with subsequent NMDAR down-regulation provide a plausible mechanism for a schizophrenia-like illness with psychiatric symptoms in NMDAR encephalitis. Likewise, neurodevelopmental effects of *in utero* exposure to NMDAR antibodies may lead to a neurodevelopmental disease phenotype through similar pathophysiological cascades. These may include recently reported short-term effects, i.e. reduced levels of cell-surface and synaptic NMDAR, increased dendritic arborization, and decreased density of mature (mushroomshaped) spines [19], and potential long-term neurodegeneration reflected by structural MRI changes with reduced volume within the midbrain, brainstem and cerebellar regions [18]. The here proposed analyses including fMRI will allow testing these hypotheses in the future, aiming to quantify the individual contributions of the various underlying mechanisms.

#### NMDAR encephalitis mouse models

In our NMDAR encephalitis model, mice were infused with a human monoclonal IgG1 antibody into the lateral ventricles which strongly bound to NMDA receptors in the brain and penetrated most parts of the mouse brain. This includes both regions that were closer to the lateral ventricles (retrosplenial cortex, hippocampal formation) and regions with further distance to the ventricle pumps (amygdala, hypothalamic regions). We assume that, in addition to the diffusion from the implantation area, antibodies also circulated and reach the brain via the systemic circulation. This would explain the strong immunostaining observed also in areas distant from the pump infusion, such as cerebellum or olfactory bulb. Visual inspection of dissected brain tissue after immunohistochemical staining confirmed the equal distribution of antibodies in the brain.

While the animals did not develop an obvious clinical phenotype, such as movement disorders or overt seizures, the seizure threshold is reduced in vivo [15]. This has recently been shown to result from reduced synaptic excitatory neurotransmission related to alterations in the dynamical behavior of brain microcircuits [62]. Thus, passive immunization of rodents with human monoclonal NR1 antibodies resulted in similar electrophysiology changes as observed in human brain tissue stemming from patients with human NMDAR encephalitis. Likewise, behavioral alterations in human patients with NMDAR encephalitis and in murine NMDAR encephalitis models showed remarkable similarities, both in passive immunization of mice using enriched human IgG from human NMDAR encephalitis patients [12] and active immunization of mice with NMDAR holoreceptors [11] or receptor peptides [13, 14]. The here observed consistent finding of impaired hippocampal connectivity in mice and in humans further suggests that NR1 autoantibodies mediate disease mechanisms in a similar way across species.

Previous studies on different NMDAR encephalitis mouse models confirmed the notion of NR1 autoantibody mediated pathology. Adult C57BL/6 mice with intrathecal NMDAR antibody exposure did not develop an overtly abnormal behavioral phenotype, but had higher epileptogenic predisposition demonstrated by increased numbers of observed convulsive seizures, a higher total seizure score, and a higher number of epileptic ‘spike’ events than the control mice [15]. Active immunization against NMDAR using an amino terminal domain peptide from the NR1 subunit resulted in a mouse model characterized by significant memory loss and decreased NMDAR cluster density in hippocampal neurons [63]. Another study reported on active immunization of immune competent mice with conformationally-stabilized, native-like NMDAR which induced a fulminant encephalitis with pronounced central nervous system (CNS) infiltration by peripheral immune cells and a phenotype that was consistent with the behavioral and pathologic characteristics of human NMDAR encephalitis [11]. However, data on detailed behavioral abnormalities, histopathological studies and further investigation of immunological mechanisms elicited by NR1 antibody exposure are required to validate the successful establishment of the various mouse models of NMDAR encephalitis. Further research is needed to determine whether active immunization [11] may better mimic the human disease compared to passive antibody transfer performed here, which might limit transferability from our findings to human disease pathology. However, as electrophysiological and functional brain changes occur down-stream of the antibody binding, passive immunization likely provides a meaningful model to study functional MRI (fMRI) changes across species.

#### Mouse model of NR1 antibody exposure *in utero*

Functional connectivity analyses were additionally carried out in a recently established murine model with *in utero* exposure to monoclonal NR1 antibodies [18]. Investigations yielded very similar functional connectivity reductions of the hippocampus in offspring at the age of 8 weeks and 10 months. Transfer of NMDAR autoantibodies from maternal mice during pregnancy via the placenta to the fetus have recently been shown to accumulate in the murine fetus leading to a considerable reduction of synaptic NMDAR density in the early neonate mouse brain. Adult NR1 offspring animals displayed an abnormal neuropsychiatric phenotype, including hyperactivity, lower anxiety and impaired sensorimotor gating persisting throughout adulthood [18]. These findings were complemented by the observation that human mothers of children with psychiatric disorders (e.g., autism spectrum disorders or schizophrenia) had higher NR1 antibody levels compared to mothers of healthy children [18]. It was hypothesized that these autoantibodies are materno-fetally transferred to the human fetus and that they contribute to a broader spectrum of behavioral abnormalities in psychiatric disorders, thus diverging from typical characteristics of adult NR1-antibody mediated disease [18].

A recently developed mouse model of placental antibody transfer used serum NMDAR antibodies representative of several patients with tail vein injection of maternal animals instead of monoclonal NR1-antibodies injected intraperitoneally, as reported herein. While analogous histopathological and neurodevelopmental changes including thinning of brain cortical layers, impairment of memory and hippocampal long-term plasticity, as well as depressive-like behavior, and poor motor coordination were observed during the first postnatal months, all clinical and pathological changes were reported to be completely reversible at the age of 4 months [19]. It was therefore suggested that diaplacentally transferred antibodies might contribute to transient effects such as the limited number of complications described in children of patients who develop NMDAR encephalitis during pregnancy [19], rather than to long-term persistent neuropsychiatric dysfunction.

Future studies using different mouse models with *in utero* exposure to antibodies are needed to elucidate the reversibility or long-term persistence of NR1-antibody pathology. However, our in vivo resting-state functional MRI (rs-fMRI) findings already provide evidence for a long-term impairment of hippocampal activity on a functional level caused by fetal NR1-antibody exposure throughout different stages of age. These findings complement structural brain changes with whole brain and regional atrophy in the same offspring NR1 mouse model that were linked to a possible NR1-antibody mediated neurodegenerative mechanism associated with a clinical phenotype. Future studies combining functional and structural MR data with neuropsychiatric features will help to further elucidate the pathophysiological mechanisms in this gestational mouse model of maternal NMDAR antibodies. In the future, similar studies should be expanded to other autoantibodies, e.g. Contactin-associated protein-like 2 (Caspr2), for which a neurodevelopmental phenotype with marked social interaction deficits after materno-fetal antibody transfer was recently suggested [64].

#### Translational applicability of resting-state fMRI

Preclinical investigations using rs-fMRI in murine disease models offer the possibility to non-invasively investigate the determinants of altered functional network signatures observed in human studies. These include associations between behavioral changes and abnormalities detected using structural MRI (i.e. T2-hyperintense lesions, atrophy) as well as histopathological correlates [20]. Limiting factors inherent to mouse rs-fMRI at 7 Tesla (T) include effects of anesthesia on brain activity and vasculature [22], and challenges in maintaining constant animal physiology throughout the entire MR investigation [65]. Despite these limitations, mouse rs-fMRI has become a reliable and valuable tool to unravel pathophysiological mechanisms in neuropsychiatric diseases – in part due to improvement of anesthesia protocols and continuous vital parameter monitoring including temperature and breathing rate measurement as performed in our study. Previous studies have shown highly reproducible large-scale networks in multi-center comparisons [20] and have provided promising accounts of translational functional network analyses in neuropsychiatric disorders such as schizophrenia [66], depression [67], autism spectrum disorder [22] and Alzheimer’s disease [68] (for further extensive reviews see Chuang and Nasrallah 2017 and Pan et al. 2015 [22, 65]). These observations add to the growing translational evidence of functional network alterations in neuropsychiatric diseases and provide a promising avenue for future comparative studies in NMDAR encephalitis patients and animal models of the disease. Aims of this approach include the identification of biomarkers for the assessment of preclinical treatment responses as well as comparative analyses of MRI alterations with histopathologic findings. In line with previous studies that investigated rs-fMRI in mouse models in other neuropsychiatric disorders such as Huntington’s disease [69] and Fragile-X syndrome [70], our findings suggest that functional connectivity may eventually allow for non-invasive monitoring of disease activity and effects of pharmacological interventions in NR1 autoimmune encephalitis mouse models. Hence, rs-fMRI functional connectivity, as a surrogate marker for brain network integrity, could complement behavioral assays – which suffer from limited translational validity – in future drug development [70].

### Limitations

We did not directly assess the presence of behavioral abnormalities in the mice investigated in our study. Consequently, the potential associations of the functional connectivity alterations with behavioral abnormalities and their pathophysiological significance await confirmation in future studies. However, it is important to note that adult C57BL/6 mice with intrathecal NMDAR antibody exposure did not develop an overtly abnormal behavioral phenotype, including studies using the identical human NR1 antibody clone [15, 62], but had higher epileptogenic predisposition. Of note, MRI investigations in our NR1-offspring mice (cohort 2 and cohort 3) are based on the same mice that have recently been described in detail [18]. The offspring had impaired sensorimotor gating during adolescence and adulthood. Cognitive assessment, and especially assessment of learning and memory (i.e. hippocampal functions), were not the main focus of behavioral assessment, thus preventing detailed correlation analyses [18]. Future research efforts will therefore encompass NR1 antibody mouse models with larger sample sizes and extensive behavioral and cognitive testing batteries to investigate correlations between phenotypic abnormalities and MR functional connectivity changes.

## Conclusion

Our study demonstrates the feasibility and validity of fMRI-based functional connectivity analyses in NMDAR antibody-mediated murine disease models and reveals a selectively impaired functional connectivity of the hippocampus. This finding mirrors observations in human patients and represents the first translational imaging biomarker of NMDAR encephalitis. Our investigations in mice with *in utero* exposure to NR1 antibodies underline the possibility to experimentally explore antibody-mediated mechanisms underlying neuropsychiatric symptomatology in constellations that are only limitedly or not at all accessible to human studies. The aim of these studies is to investigate the potential of rs-fMRI as animal and human imaging marker that may enable investigations at the interface of pre-clinical and clinical research. Furthermore, our study provides an important foundation for future comparative structural and functional MRI analyses in monoclonal NR1 antibody-based animal models and human patients, including interventional studies. These translational investigations will combine structural and functional MRI analyses, including detailed regional and large-scale network analyses, with histopathologic studies to further unravel the pathophysiology of NMDAR encephalitis, e.g. the mechanisms involved in hippocampal functional connectivity alterations and in structural (e.g. white matter) brain damage.

### Supplementary information

Supplementary Material

Supplementary Figure S1

Supplementary Figure S2

Supplementary Figure S3
