# Supplementary material for: Impaired functional connectivity of the hippocampus in translational murine models of NMDA-receptor antibody associated neuropsychiatric pathology
Source: Mol Psychiatry. 2023 Oct 24;29(1):85–96. doi: 10.1038/s41380-023-02303-9 (PMC11078734; doi:10.1038/s41380-023-02303-9)
Supplement: Supplementary file 1 — Supplementary Material [file 41380_2023_2303_MOESM1_ESM.docx]

## Supplementary Material –

## “Impaired functional connectivity of the hippocampus in translational murine models of NMDA-receptor antibody associated neuropsychiatric pathology”

### **Supplementary Figure Legends**

**Supplementary Figure S1.** Comparison of functional connectivity parameter estimates in adult NR1 antibody mouse model compared to control mice.

Components assessed were localized within the following brain regions: hippocampus, amygdala, caudate nucleus/basal ganglia, somatosensory cortex, and midbrain. Statistical significance was determined using dual regression and nonparametric permutation testing (1,000 permutations) with threshold-free cluster enhancement (TFCE) as implemented in Functional Magnetic Resonance Imaging of the Brain Software Library (FSL *randomize* function (p < 0.05, familywise error [FWE]-corrected) [34] within functionally defined regions of interest. No significant differences were observed except for the left hippocampal formation.

**Supplementary Figure S2.** Comparison of functional connectivity parameter estimates in adult 8-week-old NR1 offspring mouse model compared to control mice.

Components assessed were localized within the following brain regions: hippocampus, amygdala, caudate nucleus/basal ganglia, somatosensory cortex, and midbrain. Statistical significance was determined using dual regression and nonparametric permutation testing (1,000 permutations) with threshold-free cluster enhancement (TFCE) as implemented in Functional Magnetic Resonance Imaging of the Brain Software Library (FSL *randomize* function (p < 0.05, familywise error [FWE]-corrected) [34] within functionally defined regions of interest. No significant differences were observed except for the left hippocampal formation.

**Supplementary Figure S3.** Comparison of functional connectivity parameter estimates in adult 10-month-old NR1 offspring mouse model compared to control mice.

Components assessed were localized within the following brain regions: hippocampus, amygdala, caudate nucleus/basal ganglia, somatosensory cortex, and midbrain. Statistical significance was determined using dual regression and nonparametric permutation testing (1,000 permutations) with threshold-free cluster enhancement (TFCE) as implemented in Functional Magnetic Resonance Imaging of the Brain Software Library (FSL *randomize* function (p < 0.05, familywise error [FWE]-corrected) [34] within functionally defined regions of interest. No significant differences were observed except for the left hippocampal formation.
