# Supplementary figures and images for: Impaired functional connectivity of the hippocampus in translational murine models of NMDA-receptor antibody associated neuropsychiatric pathology
Source: Mol Psychiatry. 2023 Oct 24;29(1):85–96. doi: 10.1038/s41380-023-02303-9 (PMC11078734; doi:10.1038/s41380-023-02303-9)

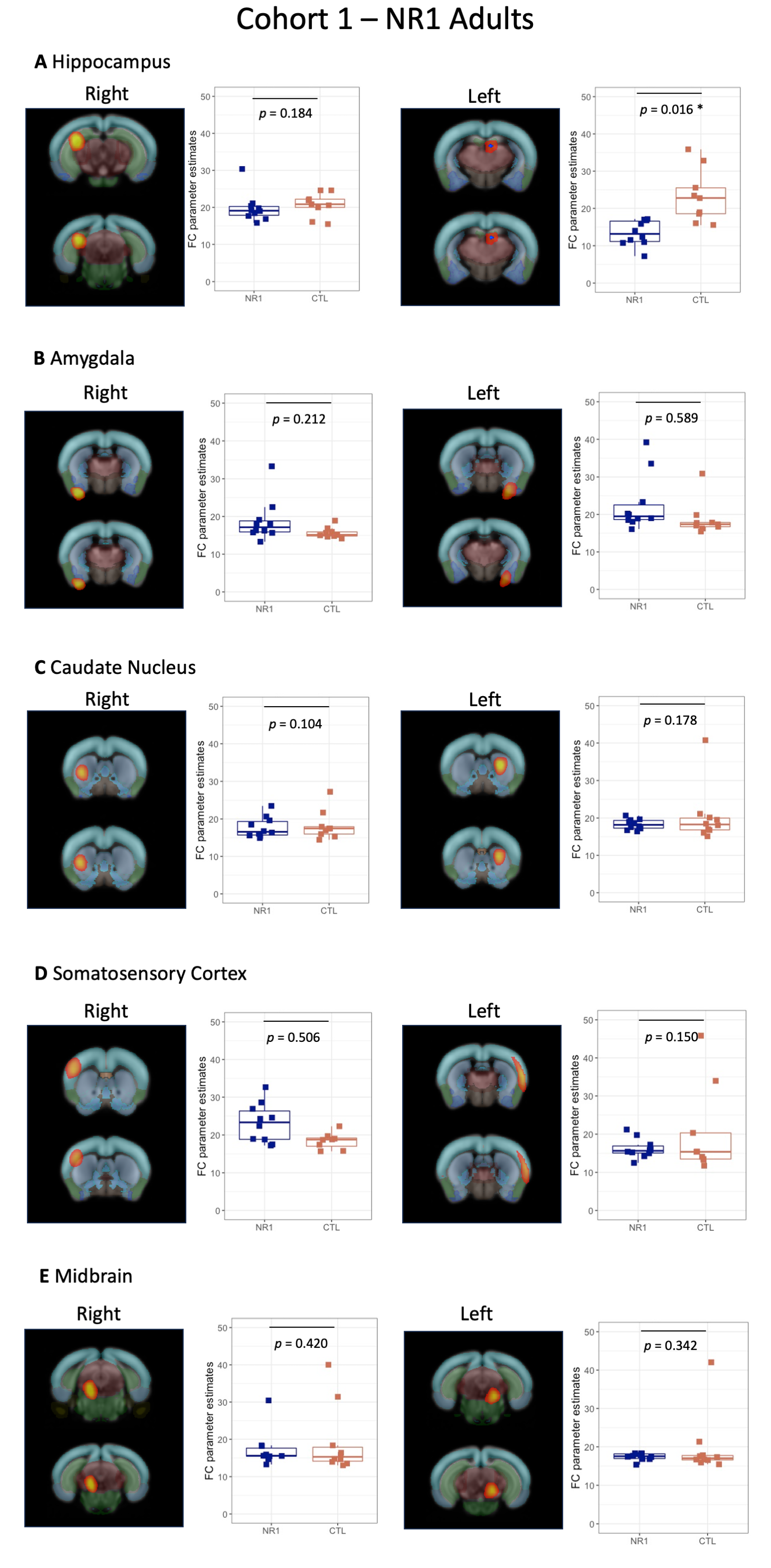

Supplement: Supplementary file 2 — Supplementary Figure S1 [file 41380_2023_2303_MOESM2_ESM.tif]

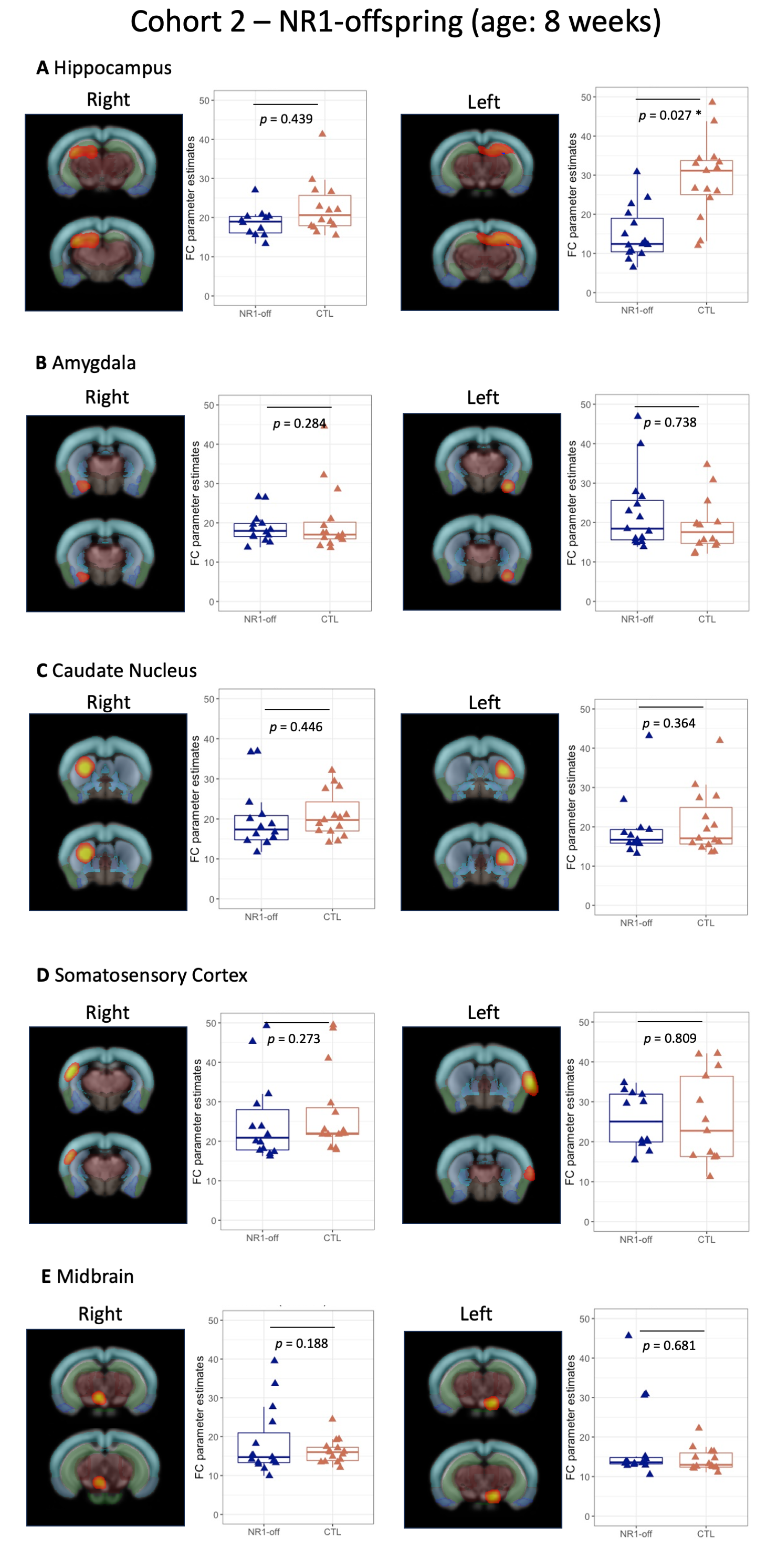

Supplement: Supplementary file 3 — Supplementary Figure S2 [file 41380_2023_2303_MOESM3_ESM.tif]

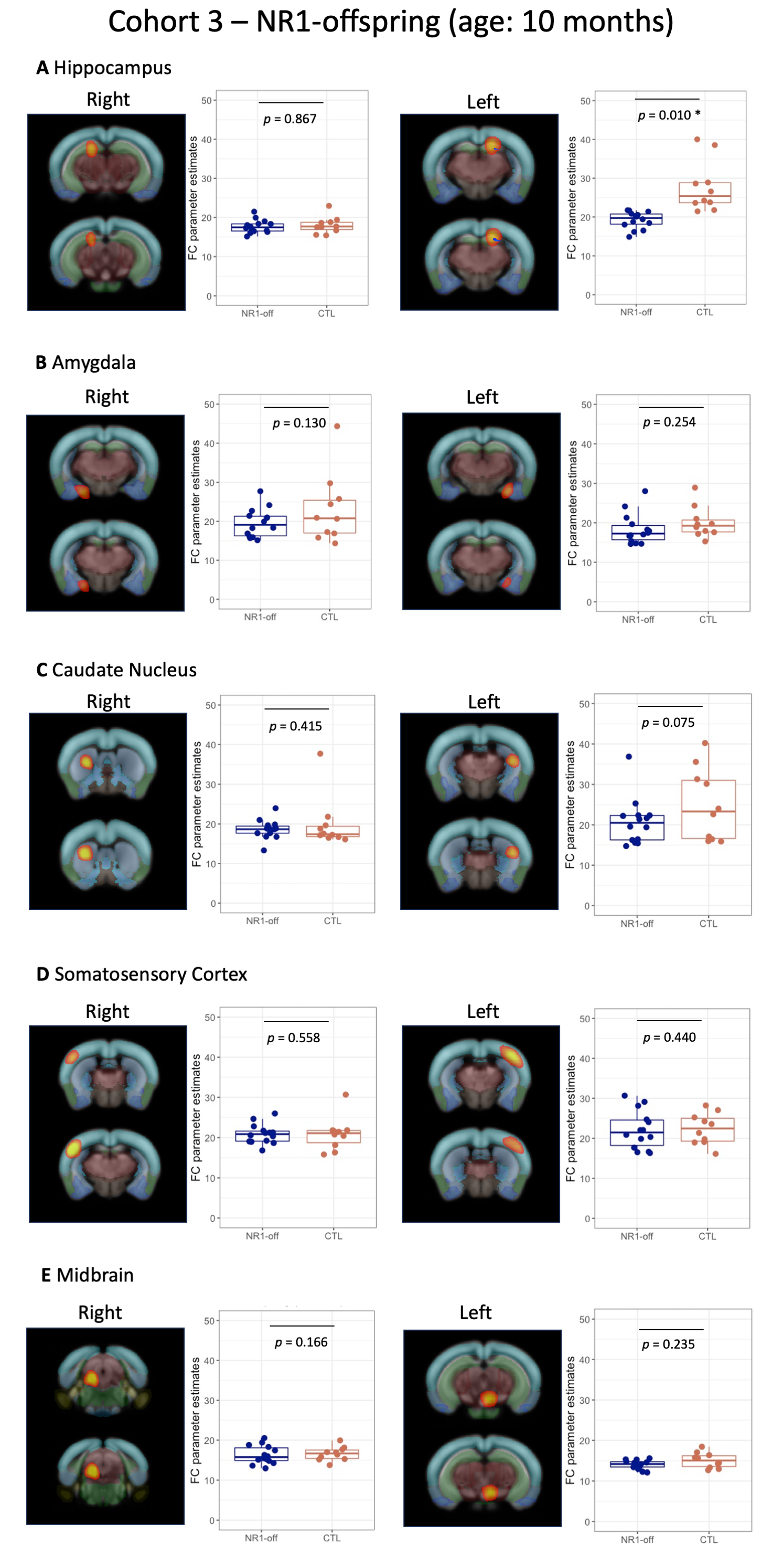

Supplement: Supplementary file 4 — Supplementary Figure S3 [file 41380_2023_2303_MOESM4_ESM.tif]
